# Parathion: MAK Value Documentation, addendum – Translation of the German version from 2024

**DOI:** 10.34865/mb5638e11_2ad

**Published:** 2026-06-30

**Authors:** Andrea Hartwig, MAK Commission

**Affiliations:** 1 Institute of Applied Biosciences, Department of Food Chemistry and Toxicology, Karlsruhe Institute of Technology (KIT) Adenauerring 20a, Building 50.41 76131 Karlsruhe Germany; 2 Permanent Senate Commission for the Investigation of Health Hazards of Chemical Compounds in the Work Area, Deutsche Forschungsgemeinschaft, Kennedyallee 40, 53175 Bonn, Germany. Further information: Permanent Senate Commission for the Investigation of Health Hazards of Chemical Compounds in the Work Area | DFG

**Keywords:** parathion, insecticide, pesticide, toxicity, evaluation, acetylcholinesterase inhibitor

## Abstract

Parathion [56-38-2] is used as an insecticide and acaricide. It is no longer approved in the European Union. The previous MAK Value documentation and addenda do not reflect the current data situation of the substance. The MAK Commissiondecided that a new evaluation is not of high priority. The MAK value and the other classifications are therefore suspended and the substance is listed in the Section II c of the List of MAK and BAT Values for substances no longer evaluated.

**Table:** 

**MAK value**	**see Section II c of the List of MAK and BAT Values**
**Peak limitation**	**–**
	
**Absorption through the skin**	**–**
**Sensitization**	**–**
**Carcinogenicity**	**–**
**Prenatal toxicity**	**–**
**Germ cell mutagenicity**	**–**
	
**BLW (2023)**	**reduction of the acetylcholinesterase activity in erythrocytes to 70% of the reference value^a)^**
	
Synonyms	*O*,*O*-diethyl *O*-(4-nitrophenyl) thiophosphate
Chemical name (IUPAC)	diethoxy-(4-nitrophenoxy)-sulfanylidene-λ^5^-phosphane
CAS number	56-38-2
Molar mass	291.26 g/mol
Melting point	6.1 °C (IFA 2023)
Boiling point	375 °C
Density at 25 °C	1.26 g/cm^3^ (IFA 2023)
Vapour pressure at 20 °C	8.9 × 10^–6^ hPa (NCBI 2023)
log K_OW_	3.83 (IFA 2023)
Solubility at 20 °C	12.4 mg/l water (IFA 2023)
**1 ml/m^3^(ppm) ≙ 12.086 mg/m^3^**	**1 mg/m^3^ ≙ 0.0827 ml/m^3^ (ppm)**

^a)^ The BLW (biological guidance value) is derived as the ceiling value because of acute toxic effects.

This addendum was prepared because the previous evaluations no longer reflect the data currently available for the MAK value and for the designations and classifications of the substance. Parathion is used as a broad-spectrum insecticide and acaricide against sucking and biting insects and mites (AERU 2022). The organophosphate acts as a strong cholinesterase inhibitor. The biological guidance value (BLW) for acetylcholinesterase inhibitors (reduction of the acetylcholinesterase activity in erythrocytes to 70% of the reference value; Lewalter 1995; Weistenhöfer et al. 2024) therefore applies to parathion. The BLW is derived as the ceiling value because of acute toxic effects. However, it was not investigated whether this is the most sensitive end point.

In 1958, a MAK value of 0.1 mg/m^3^ I (inhalable fraction) was set and the substance was designated with an “H” because it readily permeates the skin (Henschler 1973, available in German only). In the addendum from 2002, parathion was assigned to Peak Limitation Category II with an excursion factor of 8 (Greim 2002, available in German only). The substance was classified in Pregnancy Risk Group D in 1991. This classification was confirmed in 2007 (Greim 2007, available in German only).

In the European Union, the use of parathion is not permitted under Regulation (EC) 1107/2009 concerning the placing of plant protection products on the market (European Commission 2022 b; European Parliament and European Council 2009). In the Federal Republic of Germany, the use of parathion was approved from 1971 to 2002, in the former German Democratic Republic it was authorized until 1967 (BVL 2010). Parathion is listed in Annex I Parts 1 and 3 of the PIC Regulation (EC) No 649/2012 (European Commission 2022 a). Exports therefore require an export notification and the express consent of the importing country.

The previous evaluations (MAK value documentation and addenda) do not reflect the currently available data. However, a re-evaluation of the substance is not a priority. Therefore, the MAK value, the peak limitation and the “H” designation have been withdrawn and parathion has been allocated to Section II c of the List of MAK and BAT Values (DFG 2022). This section lists substances for which the previous MAK values, designations and classifications have been withdrawn and which are no longer being reviewed at present.
